# Assessing the organizational context for EBP implementation: the development and validity testing of the Implementation Climate Scale (ICS)

**DOI:** 10.1186/s13012-014-0157-1

**Published:** 2014-10-23

**Authors:** Mark G Ehrhart, Gregory A Aarons, Lauren R Farahnak

**Affiliations:** Department of Psychology, San Diego State University, San Diego, CA USA; Department of Psychiatry, University of California, San Diego, La Jolla, CA USA; Child and Adolescent Services Research Center, San Diego, CA USA

**Keywords:** Implementation climate, Organizational climate, Strategic climate, Evidence-based practice, Organizational context, Measurement

## Abstract

**Background:**

Although the importance of the organizational environment for implementing evidence-based practices (EBP) has been widely recognized, there are limited options for measuring implementation climate in public sector health settings. The goal of this research was to develop and test a measure of EBP implementation climate that would both capture a broad range of issues important for effective EBP implementation and be of practical use to researchers and managers seeking to understand and improve the implementation of EBPs.

**Methods:**

Participants were 630 clinicians working in 128 work groups in 32 US-based mental health agencies. Items to measure climate for EBP implementation were developed based on past literature on implementation climate and other strategic climates and in consultation with experts on the implementation of EBPs in mental health settings. The sample was randomly split at the work group level of analysis; half of the sample was used for exploratory factor analysis (EFA), and the other half was used for confirmatory factor analysis (CFA). The entire sample was utilized for additional analyses assessing the reliability, support for level of aggregation, and construct-based evidence of validity.

**Results:**

The EFA resulted in a final factor structure of six dimensions for the Implementation Climate Scale (ICS): 1) focus on EBP, 2) educational support for EBP, 3) recognition for EBP, 4) rewards for EBP, 5) selection for EBP, and 6) selection for openness. This structure was supported in the other half of the sample using CFA. Additional analyses supported the reliability and construct-based evidence of validity for the ICS, as well as the aggregation of the measure to the work group level.

**Conclusions:**

The ICS is a very brief (18 item) and pragmatic measure of a strategic climate for EBP implementation. It captures six dimensions of the organizational context that indicate to employees the extent to which their organization prioritizes and values the successful implementation of EBPs. The ICS can be used by researchers to better understand the role of the organizational context on implementation outcomes and by organizations to evaluate their current climate as they consider how to improve the likelihood of implementation success.

**Electronic supplementary material:**

The online version of this article (doi:10.1186/s13012-014-0157-1) contains supplementary material, which is available to authorized users.

## Introduction

There is an implementation science gap in the development and availability of practical, reliable, and valid measures to assess constructs likely to impact effective implementation of evidence-based health-care innovations [1-3]. One area in need of further attention is the development of targeted and psychometrically sound measures of organizational context for implementation. Much of the current research on organizational context of implementation has utilized global measures of general organizational culture or climate, which have been found to be related to clinician’s perspectives on evidence-based practice (EBP), client outcomes, and successful implementation [4-6]. Building on this research, this study describes the development of a measure of strategic organizational climate for EBP implementation, which can be used to support appropriate pre-assessment of organizational context and the development of strategies to accelerate effective implementation.

Organizational climate has been defined as “the shared meaning organizational members attach to the events, policies, practices, and procedures they experience and the behaviors they see being rewarded, supported, and expected” [7]. The research literature on organizational climate can be subdivided into those studies addressing *molar climate* and those addressing *focused climate* [7,8]. The study of molar climates attempts to capture the totality of the organizational environment [9], but molar climate measures have not been consistently related to organizational performance outcomes [10]. In contrast, studies of focused climate address the components of the organizational environment that are most relevant to achieve a specific outcome. Such climates include climates that focus on internal organizational processes, or *process climates*, and climates that focus on the strategic goals of the organization, or *strategic climates* [7]. Beginning with studies of service [11] and safety [12] climates, the study of focused climates is currently the most common approach to studying climate among organizational researchers and has expanded to a wide variety of topics. In addition, recent meta-analytic work has confirmed the strong relationships between focused climate and relevant outcomes across a number of domains, including service climate’s relationship with customer satisfaction [13], safety climate’s relationship with accidents [14,15], and justice climate’s relationship with a number of dimensions of unit effectiveness [16].

The focus of this paper is on a specific type of strategic climate, the climate for EBP implementation. The literature on strategic organizational climate suggests that management communicates what is valued in an organization through their actions, policies, practices, and processes [17]. As employees take in those various messages, they form an overall perception of the extent to which a specific strategic imperative is an organizational priority. When applied to EBP implementation, an effective “implementation climate” captures the extent to which employees perceive that the adoption, implementation, and use of an innovation such as EBP is expected, rewarded, and supported by the organization [18-20]. We define EBP implementation climate as employees’ shared perceptions of the importance of EBP implementation within the organization [21]. Thus, a climate for EBP implementation focuses on creating a fertile organizational context for putting EBPs into practice in an organization.

There are a number of mechanisms through which employees may perceive the value of successful EBP implementation in their organization. As such, multidimensional measures of the climate for EBP implementation are useful by providing detailed information about the factors that have the strongest impact on the climate’s development [7]. For instance, a multidimensional measure reveals the areas that are strengths for the organization and other areas where more attention may be warranted, recognizing that it is the concerted convergence of strategies to promote strategic goals across levels of an organization that ultimately results in the development of a positive and strong strategic climate [22]. To date, the most commonly cited measure of implementation climate from Klein and colleagues [21] focuses on employee’s general impressions of implementation climate but does not provide a more refined breakdown of climate dimensions. Their measure was also developed in the context of software implementation in manufacturing plants rather than health and allied health settings. Thus, the goal of this study was to provide a short, practical, and useful measure of EBP implementation climate that captured a variety of policies, practices, and procedures critical for successful EBP implementation in health and allied health settings.

In addition to scale development, we also sought to evaluate the construct-based evidence of validity of the scale by including measures of a number of other relevant constructs across the domains of climate, change, and implementation success. A summary of the constructs and the proposed relationships is provided in Table 1. For climate, we included a measure of another strategic climate, service climate, which evaluates the organization’s emphasis on delivering high quality services. We anticipated that these two strategic climates would be moderately to strongly associated because effective work groups would emphasize both generally delivering high quality services and specifically implementing EBP, but also that the two would not be completely overlapping because of the distinct focus of each set of items. We also included items capturing several dimensions of molar climate, including involvement, performance feedback, efficiency, formalization, and autonomy. We anticipated weak but positive correlations with these dimensions as they are related to aspects of generally effective work groups but are not implementation specific. For organizational change, we included items capturing types of change, uncertainty due to change, and readiness for change. We anticipated that climate for EBP implementation would be moderately correlated with the levels of planned change reported by employees, as EBP implementation is a specific type of planned change. Because work groups with a positive implementation climate would have a structure for implementation and an alignment of organizational processes and systems related to implementation, we anticipated that employees would experience less uncertainty about change in those environments (and thus, a weak, negative correlation between EBP implementation climate and uncertainty). For organizational readiness for change, we anticipated a weak, positive relationship; even though work groups with a positive EBP implementation climate would have demonstrated the ability to effectively address change, readiness also includes aspects of innovation and flexibility that are not central to EBP implementation. In addition, there are a number of changes that work groups can experience unrelated to EBP.

**Table 1:** **Proposed relationships for construct**-**based evidence of validity analyses**

**Construct**	**Proposed relationship with implementation climate**
Service climate	Positive, moderate/strong
Molar climate (performance feedback, efficiency, formalization, and autonomy)	Positive, weak
Planned change	Positive, moderate
Uncertainty due to change	Negative, weak
Organizational readiness for change	Positive, weak

In summary, the goal of this study was to develop a scale measuring strategic climate for EBP implementation that included the most relevant dimensions and that also was pragmatic and brief to allow for use of the scale for both research and applied purposes.

## Method

### Item generation

Possible dimensions for EBP implementation climate were generated based on a review of the literature on implementation climate [21] as well as the literatures on other strategic climates (e.g., service, safety) [12,23,24]. In addition, possible dimensions and specific items were generated through consultation with subject matter experts, including a mental health program leader and an EBP trainer and Community Development Team consultant from the California Institute for Mental Health [25]. The initial dimensions and items were then reviewed by four mental health program managers for additional feedback regarding face validity and content validity. The final set of items was then finalized, with any questions being resolved by the subject matter expert consultants. The final set of 38 items represented six potential content domains of implementation climate: (1) resources for EBP, (2) educational support for EBP, (3) focus on EBP, (4) selection for EBP, (5) recognition for EBP, and (6) rewards for EBP.

### Participants

Participants were direct service providers working for 32 mental health agencies in California and Pennsylvania. Of the 860 eligible employees working in participating agencies, 630 individuals working in 128 work groups/teams agreed to participate (response rate = 73.25%; 482 were from the California sample and 148 were from the Pennsylvania sample). A work group was defined in terms of the individuals reporting to the same supervisor, with a specific focus on the supervisor who was directly responsible for them (i.e., completed their performance evaluations). Approximately 53% of the teams provided outpatient services, 35% provided a combination of services or wraparound services, 10% provided home-based services, and less than 3% provided inpatient, school, or residential treatment. The average size of the groups was 5.0 (SD = 3.23). Overall, the sample was 76.5% female with an average age of 36.51 (SD = 9.65; range = 18–70). A summary of participants’ demographic information, including race, ethnicity, education, tenure, and position, is provided in Table 2.

**Table 2:** **Demographics of the participant sample**

**Characteristics**	**Values**
Race	
Caucasian	45.9%
African-American	18.3%
Asian-American	5.1%
Native American	0.7%
“Other”	30.1%
Ethnicity	
Hispanic	37.4%
Non-Hispanic	62.6%
Education	
No college	2.3%
Some college	7.5%
College degree	25.4%
Master’s degree	62.0%
Ph.D. or M.D.	2.9%
Gender	
Female	76.5%
Male	23.5%
Position	
Intern/trainee	43.7%
Licensed provider	16.6%
Neither	39.6%
Age	
Mean (SD)	36.51 years (9.65)
Tenure with agency	
Mean (SD)	3.32 years (2.89)
Tenure in mental health	
Mean (SD)	6.25 years (5.15)

For both the California and Pennsylvania data collection, the study was approved by the appropriate Institutional Review Boards (San Diego State University, University of California, San Diego, University of Pennsylvania, and City of Philadelphia), all participants provided consent to participate, participation was voluntary, and participants could decline or withdraw from the study at any time without any negative consequences.

### Procedures for California sample

To recruit the California participants for this study, the research team approached agency executives to provide a description of the study, to gain permission to recruit their employees, and to agree on procedures for recruitment. Based on organizational charts and contact information received from agency administration, employees were contacted to participate in the study. Data were collected using online surveys or paper-and-pencil surveys. Employees received a $15 gift certificate, and supervisors received a $30 gift certificate to a major online retailer. The survey took approximately 30–40 minutes to complete. For the online survey, each participant was e-mailed a unique username and password along with a link to the web survey. Participants who agreed to participate were able to access the survey questions. In-person data collection occurred for those work groups in which online data collection was not practical (e.g., poor internet access) or if in-person was more efficient or likely to result in higher response rates. The research team reserved an hour for data collection during a regular group meeting. Research staff handed out surveys to all eligible participants and ensured completion before providing an incentive. In all cases, the supervisor completed his/her survey in a separate location to ease any concerns from participants about the confidentiality of their responses. When in-person or online data collection was not feasible, surveys were mailed to participating agencies for dissemination and employees mailed them back to the research team in prepaid envelopes.

### Procedures for Pennsylvania sample

For the Pennsylvania sample, the measures for the present study were included in a larger study of behavioral health system change. The research team approached agency executives to provide a description of the study, to gain permission to invite staff to participate in the study, and to agree upon procedures for recruitment. Eligible employees included providers and supervisors in the child outpatient behavioral health units. Data were collected during a one-hour visit held at each agency using paper-and-pencil surveys. All individuals received a $50 check for completing the larger set of measures. In general, the survey took approximately 60 min to complete. In most cases, the research team reserved an hour for data collection as part of a meeting scheduled for this purpose. Research staff handed out surveys to all eligible participants and ensured completion before providing an incentive. In all cases, the supervisor completed his/her survey in a separate location to ease any concerns from participants about the confidentiality of their responses. When in-person data collection was not feasible (e.g., a participant was late to the meeting; someone wanted time to think about whether or not they wanted to participate), surveys were left with eligible staff and participants mailed them back to the research team.

### Measures

Because the focus of this research was the work group/team level of analysis, the referent for all of the scales listed below was the team (the term used most commonly to refer to the work group in the mental health agencies that were part of this study based on our subject matter expert feedback).

#### Implementation climate

The Implementation Climate Scale (ICS) was originally developed as a part of an NIMH measure development grant (R21MH098124, PI: Ehrhart) to assess the degree to which there is a strategic organizational climate supportive of evidence-based practice implementation. Thirty-eight items were developed and evaluated based on the development process described above. All ICS items were scored on a five-point, 0 (“not at all”) to 4 (“very great extent”) scale.

#### Service climate

Service climate was measured with eight items from Schneider and colleagues [24] (*α* = 0.90). Service climate refers to employee perceptions of the practices, procedures, and behaviors that get rewarded, supported, and expected with regard to customer service and customer service quality. The research team revised items to apply specifically to a mental health setting and to the work group-level referent of interest in this study. For example, the term “business” was changed to “team”, “manager” was changed to “supervisor”, and “customer” was changed to “client”. All service climate items were scored on a 0 (“poor”) to 4 (“excellent”) scale.

#### Molar organizational climate

The Organizational Climate Measure (OCM) [26] consists of 17 scales capturing a broad range of dimensions of molar climate. In this study, we utilized three scales from the OCM as part of the construct-based evidence of validity analyses: performance feedback (*α* = 0.84, five items), involvement (*α* = 0.81, six items), and efficiency (*α* = 0.85, four items). All OCM items were scored on a 0 (“definitely false”) to 3 (“definitely true”) scale.

#### Organizational change

Organizational change was assessed using the Perceived Organizational Change (POC) measure [27]. For the present study, we utilized the planned change (*α* = 0.77, three items) and psychological uncertainty (*α* = 0.92, four items) subscales. All POC items were scored on a 0 (“not at all”) to 6 (“a great deal”) scale.

#### Organizational readiness for change

Organizational readiness for change was assessed using the Organizational Readiness for Change (ORC; *α* = 0.89, five items) subscale [28] of the larger climate measure developed by the Texas Christian University (TCU) Institute of Behavioral Research. All ORC items were scored on a 0 (“strongly disagree”) to 4 (“strongly agree”) scale.

### Statistical analyses

The sample was randomly split so that half could be utilized for the exploratory factor analysis (EFA) and half for the confirmatory factor analysis (CFA). Because of dependencies in the data due to individuals being nested within work groups, we split the sample at the work group level within organizations. This resulted in a sample of 301 providers in 64 work groups for the EFA and 329 providers in 64 work groups for the CFA (the differences in the number of providers resulted from differences in group size across the sample).

Using IBM SPSS software, EFAs were conducted utilizing principal axis factoring with promax oblique (i.e., correlated factors) rotation. Principal axes factoring was selected because it allows for consideration of both systematic and random error [29]. The number of factors was determined based on multiple criteria including the variance accounted for by the solution, the variance accounted for by each individual factor, the interpretability of the factors, and parallel analysis [30-32]. The initial criteria for item inclusion were primary loadings above 0.40 and cross-loadings below 0.30 [29]. Because we aimed to develop a brief and pragmatic measure to maximize its usefulness in both research and practice, we subsequently evaluated items based on their relative loadings on a given factor, whether they provided relatively unique content in relation to other items, and whether they would be applicable and understandable across the broadest set of participants. Parallel analysis was based on estimation of 1,000 random data matrices values that correspond to the 95th percentile of the distribution of random data eigenvalues [33]. The random values were then compared with derived eigenvalues to determine whether the parallel analyses supported the number of factors identified in the EFA.

Once the factor structure was determined based on the EFA, it was tested in the other half of the sample using CFA. The CFA was conducted using Mplus [34] statistical software adjusting for the nested data structure using maximum likelihood estimation with robust standard errors (MLR), which appropriately adjusts standard errors and chi-square values. Missing data were imputed through full information maximum likelihood (FIML) estimation. Model fit was assessed using several empirically supported indices: the comparative fit index (CFI), the Tucker-Lewis index (TLI), the root mean square error of approximation (RMSEA), and the standardized root mean square residual (SRMR). CFI and TLI values greater than 0.90, RMSEA values less than 0.10, and SRMR values less than 0.08 indicate acceptable model fit [35]. Type II error rates tend to be low when multiple fit indices are used in studies where sample sizes are large and non-normality is limited, as in the present study [36].

We tested the internal consistency reliability of the final scales (total scale and subscales) using Cronbach’s alpha. Because the scale was intended to measure a unit-level construct (i.e., the climate of clinical work groups), additional tests were conducted to assess aggregation of the individual-level responses to the unit level. These analyses included intraclass correlations (ICCs) and the average correlation within group (*a*_*wg*(*J*)_) for each subscale. ICC(1) represents the proportion of variance within a scale that is attributed to the unit (i.e., the proportion of variance that is between units as opposed to within units). *a*_*wg*(1)_ is calculated as one minus the quotient of two times the observed variance divided by the maximum possible variance, and *a*_*wg*(*J*)_ is the sum of *a*_*wg*(1)_ values for items divided by the number of items for a scale. *a*_*wg*(*J*)_ ranges from −1.00 to 1.00, with values greater than 0.60 representing acceptable agreement and values of 0.80 and above representing strong agreement [37]. Finally, construct-based evidence of validity was assessed by computing correlations between the ICS measure (overall scale and subscales) and the climate, change, and implementation success measures.

## Results

### Exploratory factor analysis

An iterative EFA process was used in applying the criteria described above. Based on the initial factor solution, three items were removed based on statistical criteria (primary loadings less than 0.40 or cross-loadings above 0.30). In the second iteration, one additional item was removed based on statistical criteria. In the third iteration, three items were removed because of overlapping content with other items and two items were removed because of language that may be unfamiliar or vague to participants (focus on evaluation and research, ability to obtain special privileges). In the fourth iteration, four items were removed because they had the lowest loadings on their respective factors and two items were removed because of redundancy with other items. In the fifth iteration, one item was removed because it had the lowest factor loading and one item was removed because of terminology that may be vague for users. In the sixth iteration, two items were removed because of unclear language and potential lack of applicability across settings. In the seventh and final iteration, one item was removed because it had the lowest loading on its factor. In total, 20 items were removed, resulting in a final scale of 18 items loading on six factors.

The variance explained by the final EFA solution was 73.46%, and the six factors individually accounted for 41.13%, 12.62%, 9.93%, 7.08%, 6.38%, and 4.57% of the variance, respectively. In addition, the parallel analysis indicated that a six-factor solution best represented the data [28-30]. Using the rotated solution for interpretation, three items loaded onto each factor and the items had high factor loadings (see Table 3). Of the six factors, five were consistent with the original proposed dimensions and one new factor (selection for openness) emerged. Of the original proposed dimensions, the only one that was not represented in the final solution was resources for EBP. The six factors were 1) selection for openness, 2) recognition for EBP, 3) selection for EBP, 4) focus on EBP, 5) educational support for EBP, and 6) rewards for EBP. As shown in Table 4, correlations among these subscales ranged from 0.16 to 0.67 at the individual level and 0.11 to 0.71 at the group level.

**Table 3:** **EFA and CFA results for the Implementation Climate Scale**

	**EFA factor loadings**						**CFA factor loadings**
**ICS items,** **subscales,** **and total**	**1**	**2**	**3**	**4**	**5**	**6**	
1. Selection for openness							
Flexible	**0.98**	0.02	−0.08	−0.01	0.02	0.00	0.97
Adaptable	**0.98**	0.05	−0.04	−0.07	0.06	−0.04	0.94
Open to new interventions	**0.65**	−0.12	0.20	0.13	−0.11	0.07	0.65
2. Recognition for EBP							
Seen as clinical experts	0.01	**0.87**	0.00	0.02	0.01	0.00	0.88
Held in high esteem	0.00	**0.85**	0.07	0.04	−0.01	−0.07	0.84
More likely to be promoted	−0.01	**0.70**	0.06	−0.04	0.02	0.14	0.65
3. Selection for EBP							
Previously used EBP	−0.06	0.04	**1.00**	−0.12	−0.05	0.01	0.84
Formal education supporting EBP	0.06	0.04	**0.72**	0.03	0.08	−0.08	0.77
Value EBP	0.05	0.05	**0.70**	0.16	−0.01	0.02	0.91
4. Focus on EBP							
Main goal is to use EBP effectively	0.03	0.03	−0.04	**0.94**	−0.05	−0.02	0.88
Think implementation is important	0.02	−0.01	0.03	**0.85**	0.01	−0.03	0.87
Using EBP is a top priority	−0.05	0.01	−0.03	**0.79**	0.12	0.05	0.86
5. Educational support for EBP							
Conferences, workshops, or seminars	0.03	0.03	−0.03	−0.13	**0.97**	0.00	0.88
EBP trainings or in-services	−0.02	0.03	−0.07	0.21	**0.75**	−0.06	0.86
Training materials, journals, etc.	−0.01	−0.09	0.19	0.11	**0.54**	0.10	0.74
6. Rewards for EBP							
Financial incentives for use of EBP	0.03	−0.01	−0.10	0.01	−0.01	**0.91**	0.76
More likely to get a bonus/raise	−0.01	0.19	−0.09	0.00	−0.06	**0.77**	0.71
Accumulate compensated time	−0.02	−0.14	0.22	−0.02	0.10	**0.63**	0.82

Bold font for the EFA factor loadings indicates the scale on which the items load.

**Table 4:** **Individual**-**level and group**-**level ICS subscale correlation matrix for the EFA sample**

	**1**	**2**	**3**	**4**	**5**	**6**
1. Selection for openness	---	0.34**	0.53**	0.38**	0.20	0.11
2. Recognition for EBP	0.31**	---	0.67**	0.60**	0.52**	0.48**
3. Selection for EBP	0.57**	0.47**	---	0.68**	0.55**	0.36**
4. Focus on EBP	0.45**	0.48**	0.57**	---	0.71**	0.46**
5. Educational support for EBP	0.34**	0.36**	0.46**	0.62**	---	0.47**
6. Rewards for EBP	0.16**	0.49**	0.29**	0.33**	0.32**	---

Individual-level correlations (*N* = 301) are below the diagonal, and group-level correlations (*N* = 58) are above the diagonal. Only groups with two or more respondents in the group are included in the group-level correlations.

**p <0.01.

### Confirmatory factor analysis

CFA was then used to test the results from the EFA in the other half of the randomly split sample. The CFA tested a six-factor model with correlated latent factors. This model demonstrated good fit as indicated by multiple fit indicators (CFI = 0.94, TLI = 0.92, RMSEA = 0.07, SRMR = 0.06). As shown in Table 3, the standardized factor loadings ranged from 0.65 to 0.97 and all factor loadings were statistically significant (*p*’s <0.001). Based on these findings, we accepted the six-factor model without additional modification.

### Scale reliability and aggregation statistics

Table 5 shows ICS total scale and item means and SDs, the scale reliabilities, and the aggregation statistics using the full dataset. Internal consistencies for the final scales were strong (*α* = 0.81–0.91). Item analyses indicated that item-total correlations were high, ranging from 0.62 to 0.87. With regard to the aggregation statistics, the ICC(1) results ranged from 0.12 (for selection for EBP) to 0.25 (for both focus on EBP and educational support for EBP), which are considered medium to large effects [38]. The ICC(1) for the overall scale was 0.25, which indicates a large effect for work group. We next examined the average agreement within group for the scales using *a*_*wg*(*J*)_ [37]. For five of the six subscales, the *a*_*wg*(*J*)_ values were approaching 0.80, ranging from 0.75 to 0.79. The only exception was the rewards for EBP subscale, which had a lower but still acceptable *a*_*wg*(*J*)_ value of 0.65. The total ICS scale had an *a*_*wg*(*J*)_ value of 0.76. Considering the overall pattern of ICC(1) and *a*_*wg*(*J*)_ values shown in Table 5, the evidence supports considering ICS items and scales as representing unit-level (i.e., clinical work group) constructs.

**Table 5:** **Summary statistics for the ICS total scale and subscales**

**ICS total and subscales**	**Mean**	**SD**	***α***	**ICC** **(1)**	***a***_***wg(J)***_
Implementation Climate Scale total	1.93	0.73	0.91	0.25	0.76
Implementation Climate Subscales					
Selection for openness	2.79	0.91	0.91	0.15	0.78
Recognition for EBP	1.68	1.10	0.88	0.17	0.75
Selection for EBP	2.09	1.00	0.89	0.12	0.79
Focus on EBP	2.28	1.04	0.91	0.25	0.78
Educational support for EBP	2.00	1.09	0.84	0.25	0.77
Rewards for EBP	0.72	0.93	0.81	0.19	0.65

### Construct-based evidence of validity analyses

Tables 6 and 7 show the correlations for the ICS total score and its six subscales with all of the proposed validity measures at the individual and group levels, respectively. For the sake of space, we focus on the pattern of findings for the ICS total score. As predicted, service climate was moderately to strongly correlated with ICS total score at both the individual (*r* = 0.50, *p* <0.01) and group levels (*r* = 0.62, *p* <0.01). We hypothesized that the three dimensions of molar organizational climate would have weak, positive relationships with ICS. All of the dimensions had statistically significant but weak correlations with the overall ICS scale score at the individual level (performance feedback: *r* = 0.29, *p* <0.01; involvement: *r* = 0.34, *p* <0.01; efficiency: *r* = 0.15, *p* <0.01), and as similar pattern was found at the group level except the correlation with efficiency was not significant (performance feedback: *r* = 0.33, *p* <0.01; involvement: *r* = 0.38, *p* <0.01; efficiency: *r* = −0.07, *p* >0.05). As predicted, EBP implementation climate was positively related to perceptions of planned change (*r* = 0.28, *p* <0.01) and negatively related to perceptions of uncertainty (*r* = −0.30, *p* <0.01) at the individual level, with a similar pattern at the group level (planned change: *r* = 0.31, *p* <0.01; uncertainty: *r* = −0.47, *p* <0.01). Additionally, EBP implementation climate had a weak, positive relationship with organizational readiness for change at both the individual (*r* = 0.17, *p* <0.01) and group levels (*r* = 0.16, *p* >0.05); although only the individual-level correlation was significant, both were in the hypothesized direction. Overall, these results show that the ICS is moderately correlated with but distinct from a conceptually similar strategic climate (service climate) and only weakly correlated with aspects of molar organization climate and change, in line with expectations. Thus, these results provide construct-based evidence of the validity of the ICS.

**Table 6:** **Individual**-**level construct**-**based validity evidence correlations**

	**ICS Total**	**Selection for openness**	**Recognition for EBP**	**Selection for EBP**	**Focus on EBP**	**Educational support for EBP**	**Rewards for EBP**
Service climate	0.50**	0.38**	0.20**	0.47**	0.41**	0.42**	0.22**
Organizational climate							
Performance feedback	0.29**	0.38**	0.02	0.25**	0.30**	0.21**	0.04
Involvement	0.34**	0.29**	0.15**	0.41**	0.25**	0.25**	0.07
Efficiency	0.15**	0.12**	0.00	0.27**	0.17**	0.09	0.01
Planned change	0.28**	0.23**	0.17**	0.24**	0.22**	0.21**	0.07
Uncertainty	−0.30**	−0.27**	−0.13**	−0.30**	−0.24**	−0.25**	−0.04
Organizational readiness for change	0.17**	0.12**	0.28**	0.13**	0.06	0.17**	−0.03

*N* = 482.

**p <0.01.

**Table 7:** **Group**-**level construct**-**based validity evidence correlations**

	**ICS Total**	**Selection for openness**	**Recognition for EBP**	**Selection for EBP**	**Focus on EBP**	**Educational support for EBP**	**Rewards for EBP**
Service climate	0.62**	0.49**	0.39**	0.54**	0.50**	0.51**	0.43**
Organizational climate							
Performance feedback	0.33**	0.36**	0.08	0.28**	0.29**	0.28**	0.27*
Involvement	0.38**	0.39**	0.27*	0.44**	0.21*	0.31**	0.19
Efficiency	−0.07	0.18	−0.19	−0.07	0.02	−0.08	−0.14
Planned change	0.31**	0.10	0.28**	0.22*	0.31**	0.32**	0.16
Uncertainty	−0.47**	−0.43**	−0.31**	−0.49**	−0.34**	−0.39**	−0.22*
Organizational readiness for change	0.16	0.07	0.43**	0.22*	−0.01	0.20	−0.10

*N* = 92. Only groups with two or more respondents in the group are included in the group-level correlations.

*p <0.05; **p <0.01.

## Discussion

The goal of this study was to create a brief, reliable, and valid measure of strategic climate for the implementation of EBP. Based on the literature on EBP implementation, on other types of strategic climates in organizations, and on the feedback from experts on EBPs, we created items to capture the aspects of the organizational environment that are most critical in supporting efforts to implement EBP. Our exploratory factor analyses revealed six unique dimensions of climate for EBP implementation: (1) selection for openness, (2) recognition for EBP, (3) selection for EBP, (4) focus on EBP, (5) educational support for EBP, and (6) rewards for EBP. In an independent sample, this dimensional structure was supported via confirmatory factor analyses. Additional analyses revealed strong support for the reliability of the scales in the ICS and the aggregation of the ICS and its subscales to the group level of analysis. Finally, our construct-based evidence of validity analyses revealed that the ICS scale was related as expected but unique from another measure of strategic climate (service climate), measures of molar organizational climate dimensions, and measures from the domain of organizational change and organizational readiness for change. Overall, the initial evidence presented here supports the ICS as a psychometrically sound measure of EBP implementation climate. The ICS and scoring instructions can be found in Additional files 1 and 2, respectively, or may be obtained from GAA.

The development and availability of a measure of strategic climate for EBP implementation is important, particularly due to recent theoretical and empirical emphasis on the importance of the organizational context for the success of EBP implementation and sustainment [20,39-41]. Furthermore, a focus on implementation climate versus general organizational climate shifts attention to those issues specifically relevant for implementation, rather than for creating a generally positive and supportive work environment (i.e., molar organizational climate). Although there is evidence that a generally positive work environment is related to implementation outcomes [6], recent theory and research in the field of organizational climate has suggested that molar climate forms a foundation for strategic climates that are then the more proximal predictors of employee behavior, customer/client experiences, and ultimately strategic success [7].

The exploratory factor analysis resulted in two changes from our originally proposed dimensionality. First, the proposed dimension of resources for EBP was not a clearly identified factor in the EFA analysis. A review of the results revealed that the items for that dimension loaded on the same factor as another dimension of the ICS, educational support for EBP, which is not surprising because the items focused on the availability of support from others or books/manuals when here were problems, which overlaps with issues related to educational support. Because the resources for EBP items had the lowest loadings in this factor and were therefore dropped in later rounds of the factor analyses. We could have included other types of resources, such as financial resources, in our measure, although our feedback indicated that this was not an issue that was determined at the work group, so it would not be useful for assessing work group climate. Future research could assess whether there are resource issues independent of educational support and controlled at the work group level that could be included, in addition to how the implementation climate at the work group is affected by funding decisions and challenges at higher levels. Second, the original dimension of selection for EBP ended up being divided into two dimensions: selection for openness and selection for EBP. During our item development process, our subject matter experts indicated that due to the large variety of EBPs that may be in use at any given time and the unknown need for new EBPs in the future, it may be less important to select for EBPs in some situations but instead to select for those individuals who are most likely to be willing to adopt new practices if necessary (i.e., those who are adaptable, flexible, and open to adopting new interventions) [42]. Therefore, the final structure captures both the focus on EBPs specifically in selection as well as selecting for more general characteristics likely to be important for EBP adoption and use.

The pattern of findings for the rewards for EBP subscale was notably different than the other dimensions. For instance, the mean for this scale (0.72 on a 0–4 scale) was clearly lower than the other dimensions (with means ranging from 1.68 to 2.79). Thus, it is clear that providing financial incentives for EBP use is relatively uncommon in the agencies studied, most likely because of the availability of financial resources. As a result of this relatively low baseline, the correlations with the other subscales at the individual level were low (*r*’s = 0.16–0.33), with the exception of the correlation with recognition for EBP (*r* = 0.49) due to the clear conceptual connection between rewards and recognition. Although removing this subscale may have resulted in stronger fit for the overall structure of the scale, we concluded that the practical importance of its inclusion outweighed any statistical improvements that may have resulted. Specifically, our goal was for the ICS to be useful to researchers and also for organizations and purveyors implementing EBP. Even though providing tangible or fiscal rewards may not be common, we assert that it is important to consider attaching rewards for learning and using EBPs. In addition, the pattern of correlations at the group level was more encouraging, with strong significant correlations with all the other subscales (*r*’s = 0.36–0.48) except for selection for openness (*r* = 0.11). Future research should consider the importance of this particular dimension of implementation climate for implementation effectiveness.

There are several limitations and additional future directions for research using the ICS. This study focused on the implementation of EBPs in mental health agencies, but our goal is that the measure will be useful in other health-care contexts as well. Therefore, future research should examine the relevance for these scales in other settings where EBP implementation is a major focus (e.g., medical settings, substance abuse treatment, child welfare). We also focused the items in this measure on the work group level, as we have argued in our other research [43-45] for the critical role of the first-level supervisor for implementation success. Thus, the evidence would support using this measure for subunits within organizations (changing the referent to whatever is appropriate in a given context, such as group, department, etc.). However, we anticipate that the measures could be used at other levels of analysis as well, such as the service systems or organizations as a whole [46], but future research is needed to assess the validity of doing so. Related to this issue, we found small but significant mean differences between the California and Pennsylvania samples on four of the six ICS subscales as well as the ICS total score. Although such differences may reflect substantive differences between systems or agencies in the two states (particularly related to EBP funding), they may also be related procedural differences in data collection between the two, which is a limitation to the current study.

Another direction for future research is that organizations may be implementing multiple EBPs at a given time. We focused generally on EBPs in the item wording rather than on any specific EBP. In some applications, it may be useful to adapt the wording to focus on the climate for the implementation of a specific EBP. We anticipate that such an adaptation would be meaningful and empirically supported, but future research should specifically address whether this is indeed the case. Finally, we did not assess predictors of implementation climate or whether the ICS was related to actual implementation outcomes. For instance, perceptions of implementation climate may be impacted by the program’s history with EBP implementation, the stage in the implementation process, and the funding source and funding availability. In addition, although there is other evidence that molar organizational climate is related to implementation outcomes [6] and that other strategic climates are strongly related to relevant strategic outcomes [13-15], future research should specifically assess the criterion-related validity evidence for this measure.

The distinctive qualities and value of the ICS should be considered in comparison with other scales assessing various aspects of the organizational context. Perhaps the most commonly cited measure of organizational context as it relates to EBP implementation is the Organizational Social Context (OSC) measure by Glisson and colleagues [47-49]. This measure assesses the overall organizational culture along three dimensions (proficiency, rigidity, resistance) and the molar organizational climate across three dimensions (engagement, functionality, stress), as well as the overall morale of the workforce. Although these dimensions have been shown to be related to client-level outcomes [6], the measures are not focused specifically on implementation-related issues. In other words, the scales capture employee’s general perceptions of the work environment that, in line with recent theory and research on organizational climate, likely form a foundation on which strategic climates (like implementation climate) can be developed. Other relevant scales include those measuring readiness for change [28,39,50]. Such measures capture a variety of issues, including what the organization’s current needs are [28], the plan and roles for a specific intervention [50], aspects of the organization’s general culture or molar climate [28,50], or whether workers are committed to change and are confident in their ability to implement a change [39]. Although all of these issues are important, in our view, they do not capture implementation climate based on our definition of employees’ shared perceptions of the importance of EBP implementation within the organization.

The most similar measure to the ICS is the implementation climate measure recently published by Jacobs and colleagues [51] that was developed simultaneous to this one. Their measure is also a strategic climate measure focused specifically on EBP implementation. Their measure has six items capturing three dimensions: expectations, support, and rewards. A comparison of the items reveals some similarities and key differences. Both measures capture issues related to support and rewards, although the items in the ICS are focused on more specific issues related to the policies, practices, and procedures in the organization. Thus, the Jacobs et al. measure [51] is useful for measuring perceptions of the overall implementation climate in very few items. The ICS may offer different and practical benefits by providing organizations with feedback on how employees perceive very specific issues related to implementation. In addition, if only global perceptions of implementation climate are desired, then, the ICS focus on EBP subscale alone may be adequate. In summary, although we would argue for the distinctiveness of the ICS relative to these various measures of other constructs related to organizational context, more research is needed to show how they independently and collectively enhance our understanding of implementation effectiveness in service systems that are complex, multilevel, and multifaceted.

## Conclusions

The current study builds on past research on implementation climate by clarifying a number of specific issues relevant for developing a climate for EBP implementation. In particular, organizations desiring to create a climate for EBP implementation should emphasize the importance of organizational priority of EBP implementation, provide educational support for the EBPs being implemented, recognize and reward employees for successful implementation and use of EBPs, and select employees based on their openness to the use of EBPs and/or their previous experience with EBPs. The ICS allows organizations to assess employees’ perceptions of the current climate with regard to all of these issues, while also being brief enough (18 items) to be of practical use by both managers and researchers. Future research should address how strategic climate for EBP implementation interacts with other organizational factors and implementation approaches to predict employee behaviors and ultimately implementation efficiency and effectiveness.

## Additional files

Additional file 1:
**Implementation Climate Scale.**
Additional file 2:
**Implementation Climate Scale Scoring Instructions.**
