# Supplementary material for: Assessing the organizational context for EBP implementation: the development and validity testing of the Implementation Climate Scale (ICS)
Source: Implement Sci. 2014 Oct 23;9:157. doi: 10.1186/s13012-014-0157-1 (PMC4210525; doi:10.1186/s13012-014-0157-1)
Supplement: Additional file 1: — Implementation Climate Scale. [file 13012_2014_157_MOESM1_ESM.pdf]

Implementation Climate Scale (ICS)

Mark G. Ehrhart
mehrhart@mail.sdsu.edu

Gregory A. Aarons
gaarons@ucsd.edu

This 18-item measure assesses the degree to which there is a strategic organizational climate supportive of evidence-based practice implementation. Implementation climate is defined as employees' shared perceptions of the policies, practices, procedures, and behaviors that are rewarded, supported, and expected in order to facilitate effective EBP implementation.

Note: This measure can be adapted to study climate for evidence-based practice implementation for teams/work groups or entire organizations. Please choose a single referent point for all of the items (e.g., team or agency).

Instructions: Please indicate the extent to which you agree with each statement.

**0**	**1**	**2**	**3**	**4**
**Not at all**	**Slight extent**	**Moderate extent**	**Great extent**	**Very great extent**

Focus on Evidence-Based Practice

1. One of this team/agency's main goals is to use evidence-based practices effectively..... 0 1 2 3 4

2. People in this team/agency think that the implementation of evidence-based practices is important.......... 0 1 2 3 4

3. Using evidence-based practices is a top priority in this team/agency.......... 0 1 2 3 4

Educational Support for Evidence-based Practice

4. This team/agency provides conferences, workshops, or seminars focusing on evidence-based practices.......... 0 1 2 3 4

5. This team/agency provides evidence-based practice trainings or in-services.......... 0 1 2 3 4

6. This team/agency provides evidence-based practice training materials, journals, etc.... 0 1 2 3 4

Recognition for Evidence-Based Practice

7. Clinicians in this team/agency who use evidence-based practices are seen as clinical experts.......... 0 1 2 3 4

8. Clinicians who use evidence-based practices are held in high esteem in this team/agency.......... 0 1 2 3 4

9. Clinicians in this team/agency who use evidence-based practices are more likely to be promoted.......... 0 1 2 3 4

Rewards for Evidence-Based Practice

10. This team/agency provides financial incentives for the use of evidence-based practices 0 1 2 3 4

11. The better you are at using evidence-based practices, the more likely you are to get a bonus or a raise.. 0 1 2 3 4

12. This team/agency provides the ability to accumulate compensated time for the use of evidence-based practices.. 0 1 2 3 4

Selection for Evidence-Based Practice

13. This team/agency selects staff who have previously used evidence-based practice....... 0 1 2 3 4

14. This team/agency selects staff who have had formal education supporting evidence-based practice 0 1 2 3 4

15. This team/agency selects staff who value evidence-based practice................................ 0 1 2 3 4

Selection for Openness

16. This team/agency selects staff who are adaptable... 0 1 2 3 4

17. This team/agency selects staff who are flexible.. 0 1 2 3 4

18. This team/agency selects staff open to new types of interventions 0 1 2 3 4
