# Supplementary material for: Assessing the organizational context for EBP implementation: the development and validity testing of the Implementation Climate Scale (ICS)
Source: Implement Sci. 2014 Oct 23;9:157. doi: 10.1186/s13012-014-0157-1 (PMC4210525; doi:10.1186/s13012-014-0157-1)
Supplement: Additional file 2: — Implementation Climate Scale Scoring Instructions. [file 13012_2014_157_MOESM2_ESM.pdf]

Implementation Climate Scale (ICS) Scoring Instructions

Mark G. Ehrhart
mehrhart@mail.sdsu.edu

Gregory A. Aarons
gaarons@ucsd.edu

This measure assesses employees' shared perceptions of the policies, practices, procedures, and behaviors that are rewarded, supported, and expected in order to facilitate effective EBP implementation.

Abbreviated Items and Scoring

Item #	Scale	Factor Loading	α
	**Scale 1: Focus on EBP**		**.91**
1	One of this team/agency's goals is to use EBP effectively	.94	
2	People think implementation is important	.85	
3	Using EBP is a top priority in this team/agency	.79	
	**Scale 2: Educational Support for EBP**		**.84**
4	Provides conferences, workshops, or seminars	.97	
5	Provides EBP trainings or in-services	.75	
6	Provides EBP training materials, journals, etc.	.54	
	**Scale 3: Recognition for EBP**		**.88**
7	Clinicians who use EBP are seen as clinical experts	.87	
8	Clinicians who use EBP are held in high esteem	.85	
9	Clinicians who use EBP are more likely to be promoted	.70	
	**Scale 4: Rewards for EBP**		**.81**
10	Provides financial incentives for use of EBP	.91	
11	More likely to get a bonus or a raise	.77	
12	Provides ability to accumulate compensated time	.63	
	**Scale 5: Selection for EBP**		**.89**
13	Selects staff who have previously used EBP	1.0	
14	Selects staff who have formal education supporting EBP	.72	
15	Selects staff who value EBP	.70	
	**Scale 6: Selection for Openness**		**.91**
16	Selects staff who are adaptable	.98	
17	Selects staff who are flexible	.98	
18	Selects staff who are open to new types of interventions	.65	

SCORING THE SCALES

The score for each subscale is created by computing a mean score for each set of items that load on a given subscale. For example, items 1-3 constitute Scale 1.

COMPUTING THE TOTAL SCORE

A mean of the scale scores may be computed to yield the mean score for the total ICS.

Please direct any questions to Dr. Aarons by email at gaarons@ucsd.edu.
